# Acceptability and feasibility of implementing thermal ablation as a preventive cervical cancer treatment and the comparison of treatment outcome with cryotherapy in Zimbabwe

**DOI:** 10.3332/ecancer.2024.1736

**Published:** 2024-08-12

**Authors:** Malvern Munjoma, Stephano Gudukeya, Jabulani Mavudze, Charity Chipfumbu, Hanul Choi, Tafara Moga, Blessing Mutede, Staci Leuschner, Noah Taruberekera

**Affiliations:** 1 Population Solutions for Health, Harare, Zimbabwe; 2 Population Services International, Harare, Zimbabwe

**Keywords:** thermal ablation, cervical cancer screening, low-resource setting, feasibility

## Abstract

**Introduction and background:**

Thermal ablation, a technique that destroys precancerous cervical cells by extreme heat or cold, is predominantly used as a preventive cervical cancer treatment modality in high-income countries. Compared to other treatment methods thermal ablation has numerous advantages in its portability, minimal electricity use and comparable treatment rates, which is convenient for use in low- and middle-income countries (LMICs). Therefore, it is important to understand acceptability among providers and clients and the feasibility of achieving comparable treatment outcomes with other methods in LMICs.

**Methodology:**

We conducted a prospective longitudinal, open-label two-arm study from June 2021 to April 2022 at seven health delivery points. In this study, 182 clients were enrolled to receive preventive cancer treatment at baseline and followed up 6 months later to measure treatment outcomes and experiences on the procedure. Eligible consented clients were elected to a preferred method (either thermal ablation as an intervention or cryotherapy as a control group). We also conducted qualitative interviews with 14 service providers in either static or outreach settings.

**Results:**

At the 6-month follow-up, the efficacy was comparable among the two groups, 96.5% (95% CI 86.7%–99.1%) clients in the intervention group had successful lesion treatment rate compared to 80.8% (95% CI 69.9%–99.1%) of the control group. Furthermore, 99% of clients in the intervention group would recommend thermal ablation to their family members or peers. Service providers preferred thermal ablation due to its ease of use, lower costs, portability and lower likelihood of adverse events compared to cryotherapy.

**Conclusion:**

The study showed the feasibility of implementing thermal ablation as a new preventive cervical cancer treatment modality in Zimbabwe. Furthermore, service providers indicated their preference for thermal ablation due to its ease of use, portability at static settings and lower likelihood of adverse events occurrence. Therefore, we recommend scaling up thermal ablation both at static and outreach sites.

## Introduction

Over 90% of deaths from cervical cancer occur in low- and middle-income countries, and cervical cancer accounts for the top cause of mortality among women ages between 15 and 49 in Zimbabwe, accounting average of 39.2 deaths in 100,000 women per year [1]. There are multiple factors attributed to the high mortality rate due to cervical cancer in Zimbabwe, such as late diagnosis, lack of treatment equipment and lack of public awareness of prevention [2]. The Zimbabwean government developed a national screening program for cervical cancer, yet there is no guideline on the care continuum and cervical cancer management strategy [3, 4]. In 2020, Zimbabwe recorded a 0.65 cervical cancer mortality-to-incidence ratio, which urges to implementation of cost-effective, low-maintenance technology to detect and treat pre-invasive lesions at early stages [1]. The World Health Organisation (WHO) previously developed a same-day screen-and-treat approach, using HPV DNA detection as a primary screening tool and then treating if tested positive to lower patients’ burden to visit the clinic twice, and this has proven effective in treatment success rate and costs than regular cytology-based screenings [5, 6].

There are several methods to treat pre-cancerous lesions, also known as cervical intraepithelial neoplasia (CIN), in resource-limited settings. For high-grade CIN (CIN2-3), ablative (destroying abnormal tissues by exposing them to extreme temperatures using thermal coagulation or cryotherapy) or excisional methods (removing abnormal tissues through the surgical procedure) are the most widely used [7]. In 2019, the WHO recommended using thermal ablation (coagulation) or cryotherapy to treat HPV-positive women and loop electrosurgical excision procedures for those who are ineligible for ablative methods [6]. Cryotherapy involves applying a cooled metal disc to the cervix to freeze pre-cancerous tissues and can be offered immediately after the screening [6]. However, this treatment modality may not be feasible in low-resource settings due to its high maintenance costs, inconsistent supply of refrigerant gas and the immobility of the machine. For instance, less than half of eligible women received cryotherapy in Malawi due to gas stock outs, machine failures and lack of cryotherapy machines in the district [8, 9]. On the other hand, thermal coagulation uses a heated probe to burn cervical tissue to induce necrosis. While it has been primarily used in high-income countries, its portability and comparable treatment success rate motivated researchers to implement it in low-resource settings [10]. A meta-analysis of 13 studies on the treatment efficacy of thermal coagulation showed a 96% (95% CI 92%–99%) cure rate with a low side effect recorded [11]. However, there is a gap in understanding the treatment efficacy of various ablative methods in identical resource-limited settings [12].

Although there is an increased uptake of thermal coagulation after the WHO recommendations and guidelines in 2019, there is still a knowledge gap in provider preparedness and understanding of factors influencing the acceptability of this method in low- and middle-income countries (LMICs) [5]. Researchers in Asia, Africa and South America primarily evaluated cure rates on CIN grades one and two using thermocoagulation, and only two studies assessed feasibility and providers’ acceptability [13, 14]. Among patients, pain and pain management were key factors in determining the novel preventive cervical cancer treatment method’s acceptability. For instance, the study in Cameroon associated clients’ self-reported level of pain and post-procedure side effects with their level of acceptability of thermal ablation [7]. On the other hand, researchers in rural Malawi evaluated provider’s acceptability, and providers reported a greater acceptability for thermocoagulation among clients due to a shorter duration of treatment in addition to a lower level of pain during the procedure [8]. Providers appreciated the sustainability of thermocoagulation in contrast to cryotherapy, which hindered them from providing treatment services continuously [8]. Therefore, this study sought to identify the feasibility and acceptability of thermal ablation as a cervical cancer prevention method among providers and clients in Zimbabwe.

## Methods

### Study design

A prospective longitudinal, open-label two-arm study was employed to identify the acceptability and feasibility of implementing thermal ablation as a preventive cervical cancer treatment method in both static and outreach settings in Zimbabwe. We measured treatment outcomes and women’s experiences with thermal ablation procedures from June 2021 to April 2022. Women ages 25 and 59 years old who tested positive for precancerous lesions using visual inspection with acetic acid and cervicography (VIAC) and received either cryotherapy or thermal ablation as a treatment at one of seven New Start Centres (NSCs, New Africa House, Bambanani, Gweru, Mutare, Chipinge, Concession and Chitungwiza) were recruited for this study and followed up at 6 months.

A total of 182 eligible women who tested positive for precancerous lesions and were treated with either cryotherapy or thermal ablation (69 cryotherapy, 113 thermal ablation) enrolled at baseline, and 65 cryotherapy, and 58 thermal ablation clients were followed up at 6 months. Eligible clients consented and were assigned to a preferred treatment method. They were allowed to choose a method of their choice. After the ablation, we administered one-on-one exit interviews using structured questionnaires to understand their experiences. We measured pain during the procedure and treatment satisfaction on a 10-point Likert scale of 1 (no to minimal pain/best experience) to 10 (worst pain/experience). Point estimates were calculated and presented with 95% confidence intervals to assess treatment outcomes between the two methods.

We also interviewed 14 purposively selected service providers, two at each of the seven sites, who had previously performed cryotherapy at the static and outreach settings to understand providers’ perceptions of thermal ablation as a new preventive cervical cancer treatment modality. Providers did not have prior training or experience with thermal ablation, and training was conducted in 2019 at NSCs by skilled professionals.

Quantitative data were collected using Survey ToGo (Dooblo) at baseline and 6 months follow-up among all study participants and exported into STATA version 17 (StataCorp. 2021. Stata Statistical Software: Release 17. College Station, TX: StataCorp LLC.) for cleaning and analyses. We analysed study participants’ descriptive profiles by key demographics and calculated point estimates on primary study outcomes. In-depth interviews (IDIs) with providers were voice-recorded at the site, transcribed and translated into English. Thematic content analyses were used to identify themes and key study insights.

## Results

### Demographic characteristics

A total of 182 eligible women who tested positive for precancerous lesions and were treated with either cryotherapy or thermal ablation (69 cryotherapy, 113 thermal ablation) enrolled at baseline, and 123 (65 cryotherapy, 58 thermal ablation) were followed up at 6 months. The majority (60%) were married or co-habiting and had at least secondary education (88%) (Table 1).

Nearly half of them (47.4%) self-reported HIV-positive results, and nearly a third reported vaginal discharge, while 17.7% reported STI infection, and 7% had induced abortions at least once in their lifetime. The mean age at sexual debut was 19 years (minimum age 12, maximum age 35), and the median number of sexual partners was 2 (IQR: 1–4) with some participants having had as many as 40 partners. At baseline, 54.2% of clients reported prior experience with cervical cancer screening while 44.8% had positive results (Table 2).

### Participant non-sexual risk factors

**Figure 1.**
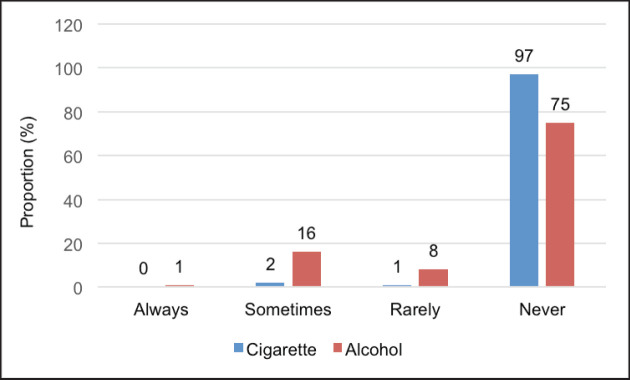
Proportion of self-reported exposure to risk factors among participants, responses were measured by the frequency of consumption of risk factors per week by the participant. ‘Always’ response refers to 7 days or more per week, ‘sometimes’ for 3–4 days per week, and rarely refers to one to twice per week.

The study also measured smoking and alcohol experiences among participants. Out of the 182 clients enrolled in the study, the majority (97%) had never smoked cigarettes while as many as 75% had never drunk alcohol (Figure 1).

### Treatment efficacy

At 6 months follow-up, successful lesion treatment outcomes were 96.5% (95% CI: 86.7%–99.1%) for clients treated by thermal ablation compared to 80.8% (95% CI: 69.9%–89.1%) among clients treated by cryotherapy. The two treatment efficacy proportions are comparable as evidenced by the overlapping confidence intervals around the point estimates.

Participants in cryotherapy reported an average pain score of 3.7 (95% CI 3.2–4.2), and the thermal ablation group reported 3.1 (95% CI 2.8–3.4) (Table 3). There was no significant difference in reported pain experience across different age groups. 4% (95% CI 1.4–12.9) of cryotherapy clients reported major bleeding occurrence compared to 11% (95% CI 6.1–17.9) of women in the thermal ablation group (Table 4). Similarly, there was no difference in reported bleeding experience between the groups. We also evaluated the acceptability of two treatment methods, and 12% of women who received cryotherapy mentioned their concerns about the effectiveness of the procedure, side effects such as vaginal discharges, and lack of sufficient counselling before receiving the treatment. On the other hand, 4% of thermal ablation clients expressed similar concerns. Overall, the mean satisfaction scores were not different between the two methods, with 8.71 (95% CI: 8.38–9.05) cryotherapy and 8.83 (95% CI: 8.58–9.09) thermal ablation. 97% of clients in the control group and 99% in the intervention group indicated that they would recommend the procedure they received to a peer.

### Provider perceptions

Furthermore, we conducted seven IDIs with nurses and five IDIs with site managers to analyse providers’ perception on thermal ablation as a cervical cancer treatment modality. In general, providers preferred thermal ablation to cryotherapy due to its portability, cost-effectiveness, simplicity of procedure and lower likelihood of side effects and adverse events. As cryotherapy requires bulky and expensive equipment, providers mentioned difficulty in maintaining consistency in offering services in outreach settings. With portable thermal ablation equipment, they experienced an increased efficiency in service delivery.

There were several barriers to thermal ablation treatment uptake among clients. First, most women did not have preferred methods due to a lack of knowledge. However, some providers mentioned that women predominantly chose thermal ablation after listening to explanations of differences between the two modalities including a shorter duration of procedure and a lower level of post-procedure pain. Providers reported misinformation among clients on new treatment modalities such as fear of infertility. The patriarchal social structure prevented them from making decisions to receive treatment without their spouses’ approval.

## Discussion

We conducted a prospective longitudinal open-label two-arm study from June 2021 to April 2022 at 7 NSCs to identify the acceptability and feasibility of implementing thermal ablation as a preventive cervical cancer treatment among women ages 25 and 59 years old who tested positive in VIAC testing at both urban and rural regions in Zimbabwe. This study showed a comparable treatment success rate among women who received thermal ablation to cryotherapy, and the perceived pain level was similar across the two groups. While some clients reported vaginal discharge after the thermal ablation procedure, there was no significant difference in adverse event incidences (bleeding, lower back pain and so on) between the two treatment modalities. Furthermore, intervention group clients had fewer concerns about the effectiveness or side effects of the treatment, and a majority of women in both groups indicated that they would recommend the treatment procedure to their peers.

Comparable health outcomes and levels of acceptability among clients were observed in other studies in LMICs. For instance, the study with women living with HIV in Western Kenya showed that most participants reported mild pain after thermal ablation at a 4–6 weeks post-treatment follow-up with no recorded severe adverse events [15]. Similarly, researchers found a high treatment efficacy rate and client satisfaction using thermal ablation 12 months post-procedure in Burundi with minimal adverse events [16]. Also, researchers in Honduras administered thermal ablation treatment to 317 women who had CIN grades 2 and 3 and a subset of grade 1 diagnoses and followed up at 12 months. Only two (0.6%) women developed invasive cancer with 75 (23.1%) at CIN2-3 [17, 18]. While 18.4% of participants reported post-procedure discomforts such as bleeding and cramping, all participants responded yes to peer recommendation [18]. A 5-year study in Cameroon demonstrated the capacity of applying same-day ‘3-T (Test-Triage-Treat) Approach’ in the low-resource setting with thermal ablation due to its quick procedure and safety [19]. This 3-T Approach using thermal ablation as the main treatment modality was adopted and validated for its safety and acceptability in many countries, such as Zambia and Malawi [20, 21], From various studies, thermal ablation has proven effective in preventing cervical cancer progression in low-resource settings.

Generally, service providers preferred thermal ablation to cryotherapy due to its ease of use, portability and lower likelihood of adverse events. Providers who operated in outreach settings highlighted its ability to facilitate consistency in service delivery as its main advantage. They noted the lack of knowledge of treatment modalities and misinformation among clients as barriers to increasing the acceptability of thermal ablation. Hence, we suggest further researching ways to combat misinformation and mobilize the community to increase treatment uptake.

## Limitations and mitigations

There were a few limitations in this study. First, intervention group participants had a high attrition rate. We followed up only 51.3% of clients in the intervention group compared to 94.2% in the control group, because most of the women who opted for thermal ablation were located in outreach settings with poor mobile connectivity for follow-ups. This attrition might lead to bias in adverse event incidence and CIN2-3 progression rate at 6 months among clients who received thermal ablation, so it is recommended to add in-person follow-ups such as home visits. Also, only seven NSCs were chosen to provide thermal ablation in this study, which may overlook demographic differences across urban and rural participants. Finally, despite some women who voluntarily chose cryotherapy over thermal ablation, we did not conduct qualitative interviews to identify their motivators or barriers to decisions that might be beneficial in creating scale-up strategies. Thus, we recommend that future researchers administer extensive qualitative interviews focusing on socio-cultural motivators and barriers to thermal ablation in low-resource settings.

## Conclusion

This research provides evidence of thermal ablation as an acceptable and feasible modality for preventive cervical cancer prevention in LMICs. Both providers and clients found it convenient and effective in outreach settings, where traditional modalities like cryotherapy had logistical challenges. We therefore recommend scaling up this intervention, particularly in low resource setting, where outreach modalities increase cancer treatment coverage.

## Conflicts of interest

The authors disclose that no conflict of interest relevant to this study.

## Funding

This study was supported by the Swedish International Development Agency (grant number: 4697), and the content is solely the responsibility of the authors and does not necessarily represent the official views of the Swedish International Development Agency.

## Ethical approval

This study was approved by the Medical Research Council of Zimbabwe (MRCZ/A/2734). All participants consented and received a copy of their informed consent form. We adhered to the national COVID-19 guidelines during data collection to minimise the risk of transmission.

## Figures and Tables

**Table 1.** Demographic characteristics at baseline (*N* = 182).

	Cryotherapy (*N* = 69, %)	Thermal ablation (*N* = 113, %)	Total (*N* = 182, %)
**Marital status**			
Married/Co-habiting	39 (56.5)	71 (62.8)	110 (60.4)
Never married	8 (11.6)	12 (10.6)	20 (11.0)
Widowed	6 (8.7)	9 (8.0)	15 (8.2)
Divorced	11 (9.7)	11 (9.7)	22 (12.1)
Separated	5 (7.2)	10 (8.8)	15 (8.2)
**Religion**			
Christianity	66 (95.6)	110 (97.3)	176 (96.7)
Traditional	1 (1.4)	1 (0.9)	2 (1.1)
None	2 (2.9)	2 (1.8)	4 (2.2)
**Education**			
Primary	6 (8.7)	15 (13.3)	21 (11.5)
Secondary	46 (66.7)	78 (69.0)	124 (68.1)
Tertiary	17 (24.6)	20 (17.7)	37 (20.3)
**Age group (years)**			
25–29	9 (13.0)	10 (8.8)	19 (10.4)
30–34	18 (26.1)	40 (35.4)	58 (31.9)
35–39	19 (27.5)	33 (29.2)	52 (28.6)
40–44	12 (17.4)	15 (13.3)	27 (14.8)
45–49	8 (11.6)	11 (9.7)	19 (10.4)
50+	3 (4.3)	4 (3.5)	7 (3.8)

**Table 2.** Clinical characteristics at baseline.

	Cryotherapy (*N* = 69, %)	Thermal ablation (*N* = 113, %)	Total (*N* = 182, %)
**HIV status**			
Positive	36 (52.2)	50 (44.3)	86 (47.3)
Negative	33 (47.8)	62 (54.9)	95 (52.2)
Status unknown	0	1 (0.9)	1 (0.5)
**Vaginal discharge**			
Yes	20 (29.0)	36 (31.9)	56 (30.7)
No	49 (71.0)	77 (68.1)	126 (69.3)
**STI infection**			
Yes	15 (21.7)	18 (15.9)	33 (18.1)
No	54 (78.3)	95 (84.1)	149 (81.9)
**Induced abortions**			
Yes	6 (8.7)	6 (5.3)	12 (6.6)
No	63 (91.3)	107 (94.7)	170 (93.4)
**Sexual debut**			
Mean age (min-max)	20 (15–30)	19 (12–35)	19 (12–35)
**Median number of sex partners**			
Median (IQR)	2 (1–4)	2 (1–4)	2 (1–4)
**Ever screened for cervical cancer**			
Yes	43 (62.3)	53 (46.9)	96(52.8)
**Screening results**			
Positive	15 (34.9)	28 (52.8)	43 (44.8)
Negative	28 (65.1)	25 (47.2)	53 (55.2)

**Table 3.** Mean pain scores by treatment method and age groups (*N* = 182).

	Cryotherapy (*N* = 69)		Thermal ablation (*N* = 113)	
**Age group**	**Mean score**	**95% CI**	**Mean score**	**95% CI**
** 25–29**	4.6	2.5–6.6	3.9	2.2–5.6
** 30–34**	3.6	2.5–4.6	2.7	2.2–3.2
** 35–39**	3.7	2.7–4.7	3.5	2.8–4.2
** 40–44**	3.2	2.0–4.3	2.8	2.0–3.6
** 45–49**	3.5	1.9–5.1	3.3	2.2–4.4
** 50+**	4.0	0.3–8.3	2.5	1.3–6.3
**Total**	3.7	3.2–4.2	3.1	2.8–3.4

Mean scores were calculated out of 10

**Table 4.** Proportion experiencing major bleeding by method.

Treatment method	Yes	No	Total	Percent yes	95% CI
**Cryotherapy**	3	66	69	4%	1.4%–12.9%
**Thermal ablation**	12	101	113	11%	6.1%–17.9%
**Total**	15	167	182	8%	

Major bleeding experience was determined by participants’ self-reporting
